# Regulation of actin cytoskeletal dynamics in T cell development and function

**DOI:** 10.3389/fimmu.2025.1622928

**Published:** 2025-06-10

**Authors:** Trang T. Lam, Mark M. W. Chong

**Affiliations:** ^1^ St Vincent’s Institute of Medical Research, Fitzroy, VIC, Australia; ^2^ Department of Medicine (St Vincent’s), University of Melbourne, Fitzroy, VIC, Australia

**Keywords:** T cells, T cell development, coronin, actin cytoskeletal dynamics, actin-binding proteins, immunological synapse

## Abstract

T cell development and function depend on precise remodeling of the actin cytoskeleton, which regulates migration, cell division, immunological synapse formation, and signal transduction. Regulators of actin include nucleators (Arp2/3, Formins) and binding proteins (coronins, cofilin, myosin) that orchestrate cytoskeletal dynamics to ensure efficient antigen recognition and signaling, while Rho GTPases (Rac1, Cdc42, RhoA) link extracellular cues to actin rearrangements, influencing both conventional T cell activation and function. Dysregulated actin dynamics contribute to immunodeficiencies and autoimmunity, and thus understanding how the actin cytoskeleton is regulated in T cells has important implications.

## Introduction

The actin cytoskeleton is a dynamic filamentous (F)-actin network that shapes T cell morphology, motility and immunological function (1, 2). Constant F-actin polymerization and depolymerization enable T cells to migrate, exit the thymus, navigate lymphoid organs, and scan antigen-presenting cells (APCs) for the recognition of antigens. Upon recognizing a peptide–Major Histocompatibility complex (MHC), a T cell forms an immunological synapse (3, 4). The mature immunological synapse consists of a central supramolecular activation clusters (cSMAC) enriched in the T cell receptor (TCR) and an integrin-rich peripheral (p)SMAC ring (5, 6). Actin polymerization is essential for assembling and maintaining this structure. TCR engagement triggers a burst of actin filament assembly that drives large-scale reorganization of the T cell lamellipodium region (7, 8). There is continuous flow of actin at the synapse. F-actin polymerizes at the periphery and flows inward – corralling TCR micro-clusters towards the center and facilitating signal amplification and APC-contact stabilization (9–12). In cytotoxic T cells, actin also facilitates directed secretion of lytic granules (1). Beyond motility, actin is important for signal transduction, converting receptor engagement into structural reorganization essential for T cell function and activation (8).

**Figure 1:**
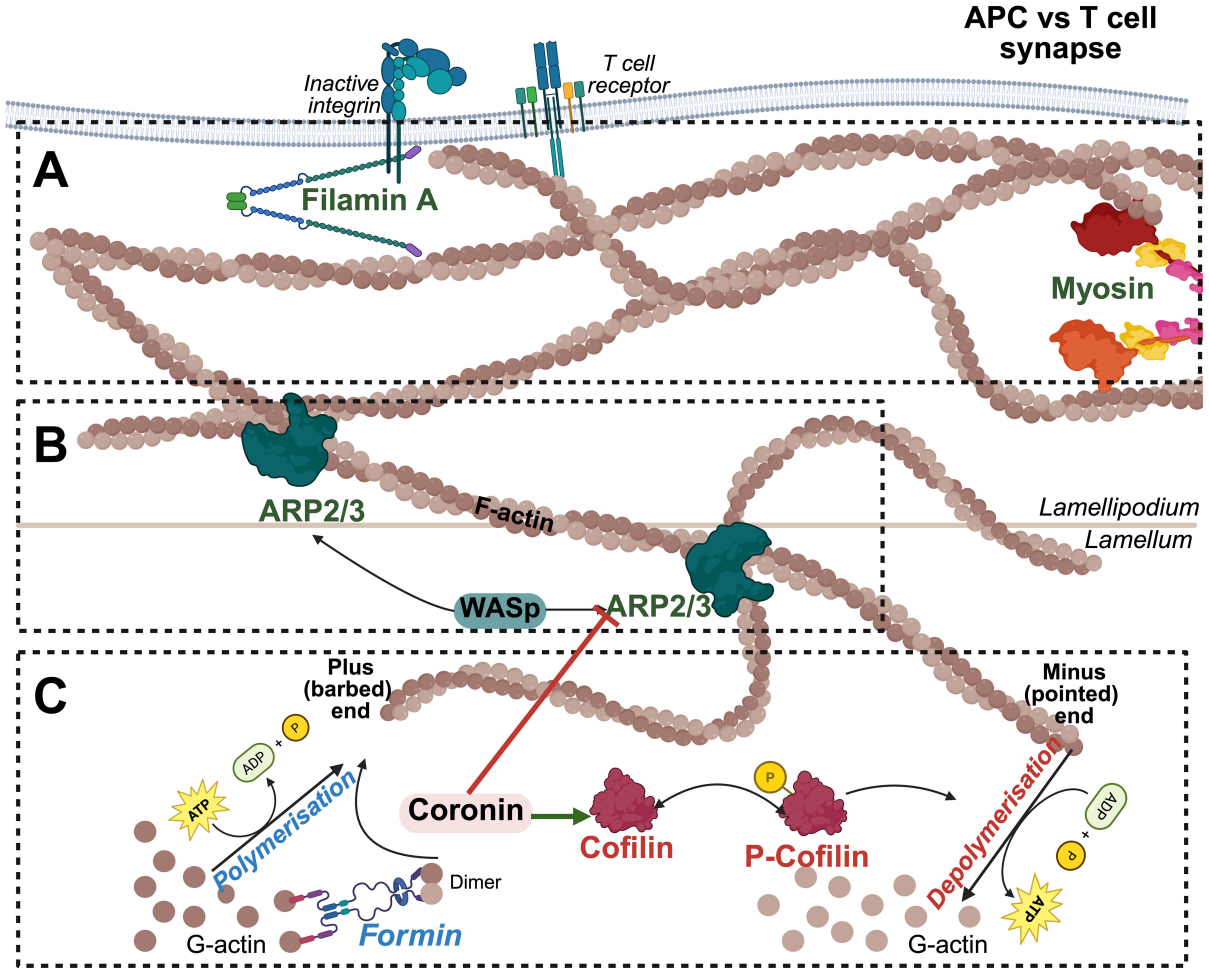
Actin Cytoskeletal Dynamics at the T Cell-APC Interface. **(A)** Actin remodeling and tension Generation: Myosin II generates contractile forces along the actin cytoskeleton, providing cortical tension necessary for immune synapse stability, T cell migration, and force-dependent signaling. Filamin A crosslinks actin filaments, stabilizing the branched architecture and reinforcing mechanical strength at T cell-APC contact site to facilitate membrane receptor anchoring. **(B)** Branched actin assembly and network stabilization: The Arp2/3 complex, activated by WASp, promotes the formation of branched actin networks that drive lamellipodia extension and immune synapse formation. **(C)** Actin polymerization and depolymerization: G-actin monomers polymerize at the plus (barbed) end in an ATP-dependent manner, a process facilitated by Formins. Formin-mediated nucleation and elongation generate linear actin filaments that serve as the foundation for cytoskeletal remodeling during T cell activation. Cofilin and coronins regulate actin filament turnover by promoting filament severing and depolymerization at the pointed ends.

Understanding the function of actin regulators is crucial for elucidating mechano-transduction in T cell biology and exploring the role of cytoskeletal dysfunction in immune tolerance and autoimmunity. T cell development and activation depend on precise actin regulation, controlled by proteins that mediate filament nucleation, stabilization, and turnover. Key regulators, including coronins, actin nucleators, actin-binding proteins and Rho GTPases, coordinate cytoskeletal remodeling for migration, synapse formation, and signaling. This review will address the roles of these regulators in T cells as highlighted in **Figure 1**.

## Actin nucleators: Arp2/3 complex and formins

T cells rely on two primary actin nucleation systems: the actin-related protein (Arp)2/3 complex, which drives branched actin polymerization, and formin proteins, which generate linear actin filaments. The Arp2/3 complex is a seven-subunit complex that binds to the side of existing “mother” filaments and initiates the growth of a new filaments at ~70° angle, thereby creating branched actin network (13). Arp2/3 on its own has low activity; it requires nucleation-promoting factors (NPFs) such as the Wiskott-Alrich Syndrome protein (WASP) and the WAVE2 complex (also called SCAR) to trigger branch formation (1, 14). T cells express a hematopoietic-specific as well as the hematopoietic WAVE complex subunit Hem-1 (Nap1) (1). Upon TCR stimulation, these NPFs are activated by upstream signals (e.g. small GTPases) and recruit Arp2/3 to form the branched actin meshwork in the lamellipodium of the immunological synapse (15, 16).

The importance of Arp2/3-mediated actin polymerization in T cell activation is highlighted by WAS, an X-linked immunodeficiency caused by WASP mutation (17, 18). Patients with WAS have defective T cell activation and cytoskeletal organization, and present with autoimmunity, highlighting the importance of actin dynamics in immune function and tolerance (18, 19). At the cellular level, WASP-deficient T cells (but not B cells) to polymerize actin at the synapse, leading to the impaired TCR clustering, PLCγ1 activation, and calcium signaling (1, 19, 20).

Similarly, mutations in ARPC1B, a core Arp2/3 subunit, results in severe immunodeficiency, autoimmunity, and defective cytotoxic function due to aberrant actin polymerization (21–23). T cells from these patients cannot generate normal lamellipodia and instead form aberrant actin spikes and filopodia, leading to unstable T cell-APC conjugates and defective cytotoxic function (23, 24). These findings underscore the importance of Arp2/3-mediated branched actin networks for T cell activation and effector functions.

In parallel, T cells rely on formin proteins (e.g., mDia1, mDia3) to generate linear actin filaments by stabilizing initial actin dimers and promoting barbed-end elongation, often producing long unbranched filaments (25). At the T cell synapse, formins cooperate with Arp2/3 to pattern the actin cytoskeleton. Live-cell imaging studies have shown that arc-like structures in the inner synapse (lamellar region) are composed of linear actin filaments generated by formin activity (26). These actin arcs are aligned concentric rings that undergo a myosin II-dependent contraction, helping to transport TCR clusters inward (26). These actomyosin arcs originate from formin-nucleated filaments behind the leading-edge synapse (25). When formin function is inhibited, these actin arcs are disorganized and TCR micro-cluster movement perturbed, indicating that formin-mediated filament elongation is required to scaffold proper synaptic architecture, Hong and colleagues also showed that the formation of *organized* actomyosin arcs depends on the strength of TCR signal and the activity of myosin II motor proteins (26). Strong agonist peptides induce robust formin-dependent arc formation, which correlates with higher mechanical forces (evidence by the tension-sensitive molecule phosphorylation) and enhanced central accumulation of signaling complexes (26). By contrast, weak TCR ligands lead to only patchy, irregular arcs. Notably, blocking myosin II contractility abrogates the difference between strong *vs* weak ligand responses, suggesting that formin-based actin structures and myosin-generated tension together amplify signals for potent antigens (25, 26). This is a form of mechano-transduction that formins facilitate. Consistently with a role in regulating the synapse, mDia1 (Diap1) knockout mice exhibit T cell activation defects, and one study found that T cells lacking both mDia1 and mDia3 had impaired division and migration (13). However, unlike Arp2/3, the role of formin proteins in T cell development has yet to be addressed.

**Figure 2:**
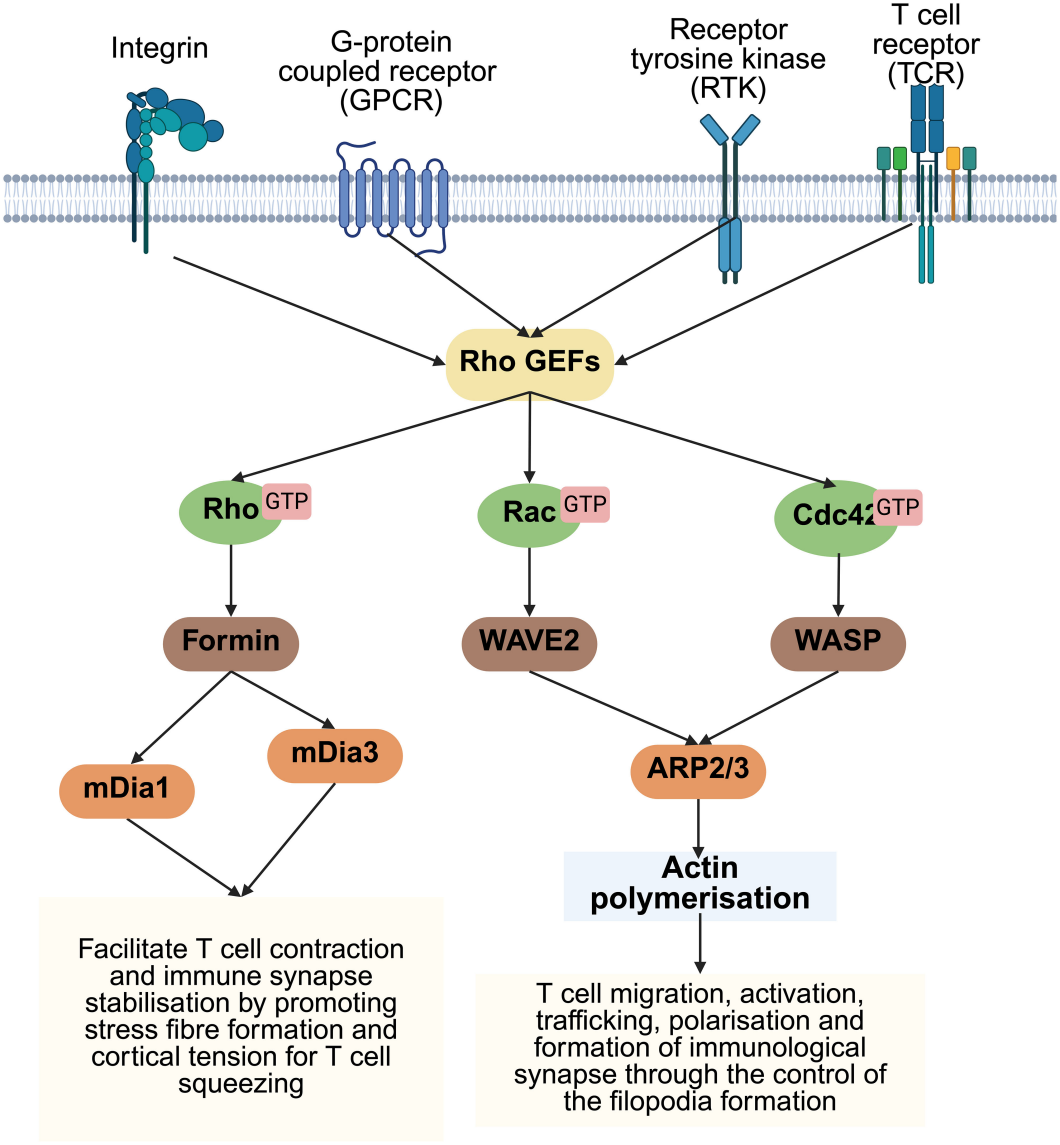
Rho GTPase-mediated signaling pathways regulating actin polymerization in T cells. Summary of the major signaling cascades initiated by surface receptors (e.g., GPCRs, cytokine receptors, TCRs) that activate Rho family GTPases via Rho GEFs. RhoA activate Formins, such as mDia1 and mDia3, to promote stress fiber formation and cortical contractility, supporting T cell squeezing and immune synapse stability. Rac1 activates WAVE2, and Cdc42 activates WASp, both of which stimulate the Arp2/3 complex, driving actin polymerization. These cascades coordinate T cell migration, activation, trafficking, polarization, and immunological synapse formation via dynamic actin remodeling.

Together, Arp2/3 and formins serve distinct yet complementary roles in T cell cytoskeletal remodeling. Arp2/3 drives broad membrane protrusions and receptors clustering, while formins stabilize actin arcs and filopodial structure. Their coordinated activity is thus essential for T cell activation, migration, and immune regulation (**Figure 2**).

Beyond the nucleation of filaments, numerous *actin-binding proteins (ABPs)* regulate filament turnover, organization, and linkage to other cellular structures. These are few exemplars with known importance in T cells: myosin motors, the filament-severing protein cofilin, the actin crosslinker filamin A, and the Ezrin-Radixin-Moesin (ERM) family that links actin to the plasma membrane.

## Myosins

Myosins are motor proteins that bind F-actin and use ATP to generate force and movement along filaments (10). T cells express non-muscle myosin II, especially Myosin IIA (encoded by MYH9), which assembles into bipolar filaments that can slide actin filaments relative to each other. In the immunological synapse, myosin II localizes to the medial zone, overlapping with the formin-derived arcs, and generates contractile tension that drives the movement of TCR micro-clusters toward the synapse center, thus sustain synapse structure (27). Kumari and colleagues showed that Myosin IIA is required for full immune synapse maturation (28). T cells lacking Myosin IIA (or treated with myosin inhibitors) formed asymmetrical or unstable synapses and had reduced central clustering of signaling molecules (28).

Myosin II also contributes to the retrograde flow of actin, where the motor pulls on filaments, adding to the inward movement initiated by polymerization at the periphery. The forces exerted by myosin II are a key part of T cell mechano-sensing, enabling the cell to probe the stiffness of the APC interface and to discriminate between strong and weak TCR signals (up to differences on the order of a few piconewtons) (26).

Besides Myosin II, T cells also have unconventional myosin, such as Myosin1g (Myo1g), which is a membrane-binding myosin implicated in forming membrane protrusions and regulating cortical tension in lymphocytes (29). Myo1g-defecient T cells exhibit altered spreading and migration, suggesting that this myosin helps T cell maintain proper cell cortex stiffness during squeezing through tissues (29, 30). Overall, myosin in T cells act as force generators and organizers, ensuring that actin filaments are appropriately positioned and tensioned for effective interaction with signaling complexes and adhesion molecules.

## Cofilin

Cofilin (also known as cofilin-1 or CFL1 in T cells) is a small protein that binds to actin filaments to promote their disassembly. Cofilin increases the off rate of actin monomers from the pointed end and can sever filaments, creating new barbed ends for polymerization. By depolymerizing actin, cofilin replenishes the pool of G-actin monomers and allows actin turnover, which is critical for dynamic processes like cell migration and synapse recycling. The importance of cofilin in T cell development was strikingly demonstrated by a genetic study in mice where *Cfl1* was replaced with a non-functional mutant specifically in T cells. This results in a severe early block in thymocyte development (31). Cofilin-deficient thymocytes were arrested at the double negative (DN) stage before successful TCRβ surface expression, leading to an absence of peripheral αβ T cells (31). These cofilin-mutant thymocytes accumulates F-actin, indicating a failure to dismantle actin structures, which likely interfered with cell division, migration within the thymus, or passage through the β-selection checkpoint. Interestingly, γδ T cells (which develop via a parallel pathway) were unaffected, highlighting that cofilin’s role is especially critical for conventional αβ T cells (31, 32).

In mature T cells, cofilin is required for effective migratory responses and synapse disassembly (33, 34). During immunological synapse formation, cofilin is initially inhibited by phosphorylation, which results in actin accumulation, but later gets activated to break down actin filaments and enable synapse dissolution and cell disengagement (10). If cofilin cannot perform this function, T cells may form overly stable contacts and fail to recycle components to interact with other APCs (35). There is also evidence that cofilin activity influences TCR signaling by facilitating actin turnover and preventing excessive F-actin build-up that could physically constrain signalosomes (36). Indeed, antigen-experienced conventional CD4 T cells have higher cofilin activity and a softer cellular cortex than naïve T cells, correlating with stronger TCR signaling (35, 37).

## Filamin A

Filamin A (FLNA) is a large actin-crosslinking protein that organizes actin filaments into orthogonal networks and links them to membrane proteins. FLNA is expressed in T cells and interacts with integrin β-chains, essentially acting as a scaffold connecting integrins like LFA-1 to actin cytoskeleton (38–40). This linkage is crucial when T cells need to apply force or resist shear stress while adhering to other cells or the endothelium. Fagerholm and colleagues found that in T cell-specific FLNA-deficient mice primary T cells, FLNA is required for optimal integrin function, which is contrary to some earlier *in vitro* findings suggesting filamin is an inhibitor of integrin activation (38, 41). *FLNA^-/-^* T cells displayed impaired adhesion under flow conditions and reduced homing to lymph nodes and sites of inflammation (38, 40). In essence, without FLNA, T cells could still activate LFA-1 to some degree but could not transmit forces effectively *in vivo* (38). FLNA tethers integrins to retrograde flowing actin, functioning as part of the “molecular clutch that allows force coupling (42). In line with this, *FLNA^-/-^* T cells have altered distribution of LFA-1 at the synapse and may form less stable immune synapse (43).

There is also evidence that FLNA can impact TCR signaling. One study reported that knocking-down FLNA impaired PKC-θ recruitment to the immunological synapse and subsequent IL-2 production, possibly because cytoskeletal anchoring of signaling complexes was affected. Notably, regulatory T cells (Tregs) express higher LFA-1 than conventional T cells and use it to form aggregates with dendritic cells (44). Tregs with compromised FLNA might not sustain these aggregates under blood flow or in the dynamic lymph node environment, potentially reducing their suppressive capacity (38, 44).

## Ezrin-Radixin-Moesin

The Ezrin-Radixin-Moesin (ERM) proteins are membrane-cytoskeletal linkers that tether actin filaments to the plasma membrane. T cells predominantly express Ezrin and Moesin (with little Radixin) (45). In resting T cells, ERM proteins are phosphorylated (active state) and help form microvilli and a rigid cortical actin shell, which maintains cell shape and spatial distribution of receptors (46). Upon T cell activation, ERMs undergo a transient dephosphorylation and re-localization, where they are briefly inactivated at the contact site to allow actin reorganization and receptor clustering and later concentrate at the distal pole complex (the face of the T cell opposite the synapse) (47). ERM proteins organize the distal pole by anchoring transmembrane proteins (like CD43 and PD-1) and preventing them from entering the synapse (47). This segregation of inhibitory or bulky molecules away from the synapse is important for efficient TCR signaling. Perturbing ERM function indeed leads to defects in T cell activation (46). For example, overexpression of dominant-negative Ezrin in Jurkat T cells causes poorly focused synapses and reduce IL-2 production (48). Conversely, T cells from Ezrin Moesin double-knockdown mice show impaired proliferation and motility (48). Upon antigen recognition, ERM proteins are rapidly inactivated via a Vav1-Rac1 signaling pathway leading to the disassociation of the cortical actin cytoskeleton from the plasma membrane (49). This un-anchoring reduces cellular rigidity, facilitating the formation of stable T cell–APC conjugates (49). Thus, ERM proteins act as architects of the T cell actin cortex, ensuring that during synapse formation certain regions of the membrane are scaffolded and others are permissive to movement.

## Coronins

Coronins are a family of WD-repeat actin-binding proteins that coordinate actin filament branching and disassembly. In mammals, Coronin-1a (CORO1A) is the best-studied member, especially in T cells where it is abundantly expressed (50–53). CORO1A localizes to F-actin-rich regions and can bind the Arp2/3 complex as well as actin filaments, positioning it to modulate actin dynamics (52, 53). Gene-knockout mouse models and the analysis of human mutations have revealed that CORO1A is essential for T cell homeostasis (54). CORO1A deficient mice have a profound T cell lymphopenia. Thymocyte development proceeds relatively normally, but thymic egress is impaired and mature naïve T cells fail to survive in the periphery (52). This phenotype mirrors a form of severe combined immunodeficiency (SCID) in humans – Immunodeficiency 8 – caused by biallelic CORO1A mutation (54). Shiow and colleagues found that a CORO1A point mutant (K26E), identified in an *N*-ethyl-*N*-nitrosourea mutagenesis screen form mice with T cell lymphopenia, disrupts thymic egress, with mutant T cells exhibiting impaired migration, abnormal actin-rich protrusions, and retention in the thymus (54). Mechanistically, this mutation enhanced CORO1A’s ability for inhibition of Arp2/3, leading to excessive actin branching that mis-localized CORO1A away from the leading edge of migrating T cells. The same study identified *Coro1A* gene mutations in a SCID patient, establishing a crucial role for T cell development in humans (51, 54). In the absence of functional CORO1A, T cells abnormally accumulate F-actin and cannot respond properly to chemokine cues for egress.

Aside from the egress defect, *Coro1a^-/-^* T cells have intrinsic signaling anomalies, exhibiting abnormal TCR-induced actin dynamics and impaired calcium and NF-κB signaling (55). CORO1A thus links cytoskeletal dynamics to TCR signaling, helping to tune the strength and duration of signals (55). Notably, CORO1A deficient T cells form overly stable synapses with prolonged contact time, correlating with hyper-accumulation of F-actin and Arp2/3 at the synapse (55). This suggests CORO1A normally promotes actin turnover at the synapse, preventing excessive actin buildup that could dampen signaling or cellular mobility.

Despite these broad peripheral T cell defects, regulatory T cells appear relatively preserved in *Coro1a^-/-^* mice. One study reported that *Coro1a^-/-^* mice were resistant to experimental autoimmune encephalomyelitis (EAE) due to loss of effector T cells, and this resistance persisted even after Treg depletion, indicating Tregs were not the main cause (56, 57). In fact, *Coro1a^-/-^* Tregs appear functionally competent *in vitro*, suggesting that primary role of CORO1A is in maintaining the naive T cell pool rather than for Treg function (57). Nonetheless, Tregs in CORO1A SCID patients have not been extensively profiled. It is possible that subtle Treg migratory or homeostatic abnormalities exist but are overshadowed by the dramatic loss of conventional T cells.

There are other members of the coronin family that may also have roles in T cells. CORO2A (also known as coronin-2 or IRF-3 binding protein) is a type II coronin and has been shown in fibroblasts to localizes to stress fibers and focal adhesions rather than the leading edge (58). In non-immune cells, CORO2A regulates focal adhesion turnover and cell motility (58, 59). Its function in T cells is less well-characterized. Type I coronins, like CORO1A, can coordinate Arp2/3 and ADF/cofilin to ensure efficient actin turnover at the leading edge (60). It will be interesting to determine if type II coronins also plays a similar regulatory role in T cell development.

## Regulatory pathways: Rho family GTPases (Rac1, Cdc42, RhoA)

Upstream of the actin regulators are signaling pathways that respond to extracellular cues to orchestrate cytoskeletal changes (**Figure 2**). Among these are the Rho family GTPases – molecular switches that cycle between an active GTP-bound state and an inactive GDP-bound state, controlling the organization of actin cytoskeleton necessary for T cell activation, polarization and migration (61). Their activation is tightly regulated by guanine nucleotide exchange factors (GEFs), which promote the exchange of GDP for GTP, and GTPase-activating proteins (GAPs), which enhance GTP hydrolysis, thereby returning them to an inactive state (62).

Rac1 is crucial for actin polymerization and lamellipodia formation, which are essential for T cell motility and immunological synapse formation (63). Upon TCR stimulation, Rac1 is activated by GEFs, such as Vav1, leading to the recruitment of WAVE2, which in turn activates the Arp2/3 complex to promote actin branching and cell spreading (14). This process is necessary for effective T cell activation, as Rac1 deficient T cells exhibit impaired TCR clustering and reduced IL-2 production (64, 65). Additionally, Rac1 facilitates T cell migration by regulating integrin-mediated adhesion and actin remodeling, which are crucial for T cell trafficking within lymphoid organs (66).

Cdc42 is another key regulator of actin dynamics and cell polarity in T cells. It controls filopodia formation, which enhances cell migration and antigen scanning (67). Cdc42 interacts with the Par3/Par6 polarity complex and atypical protein kinase C (aPKC), establishing front-rear polarity during T cell migration (68). It also plays a role in centrosome reorientation and TCR clustering at the immunological synapse, ensuring efficient signaling and sustained T cell activation (2). Loss of Cdc42 impairs the ability of T cells to form stable interactions with antigen-presenting cells (APCs), leading to defective immune responses (69).

RhoA primarily regulates actomyosin contractility through the Rho-associated kinase (ROCK) pathway, facilitating T cell contraction and immune synapse stabilization (70). It promotes stress fiber formation and cortical tension, which are necessary for T cell squeezing through tight endothelial barriers during migration (71). RhoA also controls the phosphorylation of myosin light chain, contributing to T cell shape changes required for trans-endothelial migration (71, 72). Additionally, RhoA signaling is critical for maintaining the stability of the immunological synapse, ensuring prolonged TCR signaling and proper effector function (72, 73). Rac1 and Cdc42 generally promote actin polymerization and membrane protrusions, whereas RhoA modulates actomyosin contractility and synapse stabilization (74, 75). Dysregulation of these pathways has been implicated in immune deficiencies and autoimmunity, underscoring the importance of Rho GTPase-mediated cytoskeletal control in T cell biology (74, 75).

## Concluding comments

Actin-binding proteins work in concert to shape the cytoskeleton throughout both T cell development and mature T cell function. These proteins are essential for processes such as migration, thymic selection, immune synapse formation, and signal transduction. In mature T cells, dynamic actin remodeling supports polarization, effector function, and trafficking within tissues. Despite advances in understanding these mechanisms, important questions remain, particularly regarding how specific cytoskeletal regulators coordinate with signaling pathways at distinct stages and how these dynamics influence long-term T cell fate and function. Further research is needed to fully define the roles of actin regulators across the T cell lifespan.
